# Multidisciplinary approach to treatment with immune checkpoint inhibitors in patients with HIV, tuberculosis, or underlying autoimmune diseases

**DOI:** 10.3389/fmed.2022.875910

**Published:** 2022-07-15

**Authors:** Juan Aguilar-Company, Maria A. Lopez-Olivo, Isabel Ruiz-Camps

**Affiliations:** ^1^Medical Oncology Department, Vall d'Hebron Hospital Universitari and Vall d'Hebron Institute of Oncology (VHIO), Barcelona, Spain; ^2^Infectious Diseases Department, Vall d'Hebron Hospital Universitari, Barcelona, Spain; ^3^Department of Health Services Research, The University of Texas MD Anderson Cancer Center, Houston, TX, United States; ^4^Red Española de Investigación en Patología Infecciosa (REIPI), Instituto de Salud Carlos III, Madrid, Spain; ^5^Centro de Investigación en Red de Enfermedades Infecciosas (CIBERINFEC), Instituto de Salud Carlos III, Madrid, Spain

**Keywords:** checkpoint inhibition therapy, cancer, human immunodeficiency virus (HIV), tuberculosis, autoimmune diseases

## Abstract

We reviewed the available information on the use of immune checkpoint inhibitors (ICIs) in populations with special conditions, namely, patients with HIV, tuberculosis, or underlying autoimmune disease. Available data show that treatment with ICIs is safe in patients with HIV; it is advisable, however, that these patients receive adequate antiretroviral therapy and have an undetectable viral load before ICIs are initiated. Tuberculosis reactivation has been reported with the use of ICIs, possibly due to immune dysregulation. Tuberculosis has also been associated with the use of immunosuppressors to treat immune-related adverse events (irAEs). Active tuberculosis must be ruled out in patients with symptoms or signs, and selected patients may benefit from screening for latent tuberculosis infection, although more data are required. Limited data exist regarding the safety of ICIs in patients with cancer and autoimmune disease. Data from observational studies suggest that up to 29% of patients with a preexisting autoimmune disease treated with an ICI present with an autoimmune disease flare, and 30% present with a *de novo* irAE of any type. The frequency of flares appears to differ according to the type of ICI received, with higher rates associated with PD-1/PD-L1 inhibitors. The most common autoimmune diseases for which patients reported flares with ICI therapy are rheumatoid arthritis, other inflammatory arthritis, and psoriasis. Most studies have reported flares or *de novo* irAEs associated with ICIs that were mild to moderate, with low rates of discontinuation and no deaths due to flares. Therefore, the use of ICIs in these patients is possible, but careful monitoring is required.

## Introduction

Immunotherapy has revolutionized the treatment of cancer, changing the prognosis of several tumor types. Immune checkpoint inhibitors (ICIs) act by blocking immune tolerance pathways such as programmed cell death protein 1 (PD-1), programmed death-ligand 1 (PD-L1), or cytotoxic T-lymphocyte antigen 4 (CTLA 4) and helping the immune system to recognize and attack tumor cells; however, cross-reactivity with self-proteins may cause immune-related adverse events (irAEs). irAEs can range from mild to severe or even fatal and can affect any organ system, causing a myriad of symptoms depending on the organ affected. The frequency of occurrence of irAEs differs by the type of ICI used and the characteristics of the patient. For example, treatment with PD1/PDL-1 inhibitors is associated with a lower incidence of irAEs than anti-CTLA-4 antibodies or the combination of agents of both classes (1). It is commonly believed that irAEs result from the autoreactive immune response against non-cancerous cells. To date, most clinical trials have excluded patients with underlying comorbidities such as chronic and opportunistic infections and autoimmune diseases. In patients with human immunodeficiency virus (HIV), there is concern that checkpoint inhibitors may interfere with lymphocyte function and viral suppression. Tuberculosis reactivation has been described in patients under treatment with checkpoint inhibitors, which may be related to the disruption of immune homeostasis. Patients with underlying autoimmune diseases have a higher risk of developing flares after the initiation of ICI treatment. Retrospective data suggest that the incidence of flares in these populations is substantial. Therefore, treatment with ICIs in people with cancer and underlying comorbidities needs to be approached with caution. Patients with chronic and opportunistic infections and autoimmune diseases may be difficult to treat.

In this review, we briefly summarize the current data on ICIs in patients with cancer and underlying comorbidities, specifically HIV infection, tuberculosis, and preexisting autoimmune conditions. We also include key recommendations for the management of these populations. Supplementary Table S1 summarizes the potential compli-cations associated with these three comorbidities and recommendations for managing them.

## Immune checkpoint inhibitors in patients with HIV

The life expectancy of patients with HIV receiving antiretroviral therapy (ART) is close to that observed in non-infected people. However, the chronic inflammation status of these patients leads to a higher risk of cancer and other diseases. Specifically, the risk of cancer is estimated to be 69% higher in people living with HIV than in the HIV-negative population (2). The most frequently reported neoplasms in people living with HIV are B-cell non-Hodgkin lymphoma, lung cancer, head and neck squamous cell carcinoma, Kaposi sarcoma, squamous cell skin cancer, classic Hodgkin lymphoma, and hepatocellular carcinoma (3). Cancer in people with HIV usually presents at a younger age and has more aggressive features and poorer outcomes than cancer in the general population (4). Moreover, cancer is one of the leading causes of death among people with HIV (5, 6).

Until recently, people with HIV have been excluded from clinical trials evaluating the safety and efficacy of ICIs in patients with cancer (7). This was due to concerns about the unknown effects of immunotherapy on the T-cell repertoire, the potential exacerbation of immune reconstitution syndrome in patients who recently started ART, pharmacological interactions, the possibility of unmasking opportunistic infections, and the hypothesis that people with HIV may not have sufficient T-cell immunity to benefit from PD-1/PD-L1 blockade (8, 9).

Nevertheless, treatment with ICIs may result in a dual benefit by acting on both the HIV reservoir and the cancer. PD-1-expressing CD4+ T cells constitute a known reservoir of HIV-latent infection; if immune checkpoints play a relevant role in HIV latency, ICIs could potentially improve T-cell responses against HIV antigens (10). Of note, anti-PD-1/PD-L1 treatment is effective in enhancing the production of cytokines such as IFN-γ, TNF-α, and IL-13 in response to HIV antigens (11).

### Efficacy and safety of ICIs in patients with HIV

Recently, several clinical trials involving the use of ICIs in people with HIV were reported (12, 13). In a phase 1 study including 6 patients with HIV and no other comorbidities, a single dose of the PD-L1 inhibitor BMS-936559 exhibited a good safety profile, with only grade 1 or 2 adverse events in 3 patients. An increase in HIV-specific CD8+ T cells was observed in 2 patients (12).

Another phase 1 study sought to assess the safety of pembrolizumab in advanced cancer patients with adequately controlled HIV. Thirty patients (6 with Kaposi sarcoma, 5 with non-Hodgkin lymphoma, and 19 with non-AIDS-defining cancer) were enrolled. Grade 1 or 2 irAEs were recorded in 22 patients, and grade 3 irAEs in 6 patients. HIV remained adequately controlled in all patients. As for efficacy, a complete response was observed in 1 patient, partial responses in 2 patients, stable disease in 17 patients, and progressive disease in 8 patients, with 2 patients being not evaluable (13).

A sizeable number of retrospective and prospective cohort analyses, case reports, and literature reviews have suggested acceptable safety and activity of ICIs in people with HIV, similar to findings in non-infected individuals (9, 14–18). Specifically, the incidence of irAEs does not seem to be increased and virological assessments showed that plasmatic viral load remained suppressed; however, the number of patients included in these studies was small (17).

### Recommendations

The Advisory Committee of Spanish Melanoma Group recently reviewed available data and made recommendations for the treatment and monitoring of melanoma patients with HIV who receive ICIs (19), summarized as follows: ICIs should be administered in people with HIV when the HIV viral load is undetectable and in patients receiving ART who have CD4+ T-cell counts ideally above 200 cells per mm^3^. Patients with a recent HIV-1 diagnosis should be started on ART before ICI treatment is started; viral suppression is generally achieved 4 weeks after the initiation of ART. In cases in which anticancer treatment cannot be deferred, simultaneous initiation of ICIs and ART could be considered, after assessing risks and benefits.

Before ICI treatment is initiated, screening for latent infections (including viral hepatitis, syphilis, and tuberculosis) should be performed and the infection adequately treated (20, 21). During ICI treatment, the patient should be monitored by an infectious disease specialist, ART should be continued uninterruptedly, and CD4+ cell count and HIV viral load should be periodically monitored. Transitory detectable HIV viral loads below 400 copies/ml (blips) are frequent, have no clinical significance, and require no further action. If the viral load is detected in further consecutive analyses, then additional drug resistance genotypic testing and/or drug monitoring should be performed (15).

It should be noted that the certainty of the evidence upon which these recommendations are based is low and thus the strength of the recommendations is weak. Further randomized controlled trials should confirm these recommendations.

### Conclusions

In summary, the evidence suggests that ICIs have a safety and effectivity profile in patients with HIV that is similar to that in the general population. Careful management, including a multidisciplinary approach by a team of oncologists and infectious disease specialists, is advisable.

## Immune checkpoint inhibitors in patients with tuberculosis

Tuberculosis is one of the most common infectious diseases worldwide, with about a quarter of the world's population infected with *Mycobacterium tuberculosis*, and it is one of the leading causes of death by an infectious disease. Cancer patients have an increased risk of developing active tuberculosis, and this risk is higher among patients with hematological, head and neck, and lung neoplasms (22, 23).

Although the characteristics of the interaction between the disruption of immune homeostasis caused by ICIs and tuberculosis infection are not fully understood, basic research data suggest that the PD1/PD-L1 pathway may play a substantial role in tuberculosis pathophysiology. Several underlying mechanisms have been described. PD1/PD-L1 deficiency has been associated with an increase in TNF-α, IL-1, and IFNγ (24–26) and dysregulation of the innate immune system, including macrophage and natural killer cell function (27, 28). These data suggest that downregulation of the PD-L1/PD-1 pathway induces an exacerbated inflammatory response that may facilitate the development of symptomatic infection.

In addition, patients treated with ICIs may develop irAEs, for which corticosteroids and TNF-α inhibitors could be prescribed. These therapies, especially TNF-α inhibitors, have been associated with an increased risk of developing active tuberculosis (29, 30).

### Effects of ICIs in patients with tuberculosis

Shortly after the introduction of ICIs, cases of tuberculosis reactivation and primary tuberculosis infection following the use of these agents started to be reported (31, 32). Most of the patients in whom tuberculosis was diagnosed received antituberculous treatment, and the course of the infection did not differ, in general terms, from that in patients with tuberculosis and underlying malignancy not treated with ICIs.

### Recommendations

There is an urgent need for prospective studies to validate appropriate screening and treatment strategies for ICI-related tuberculosis. Current recommendations for the clinical management of tuberculosis in patients treated with ICIs include the following: screening for latent tuberculosis infection before the initiation of ICIs, managing latent tuberculosis infection in these situations, and diagnosing and treating active tuberculosis in patients receiving ICIs. Here, we provide suggestions for clinical practice based on current evidence and our experience.

#### Screening for latent tuberculosis infection before initiation of ICIs

Latent tuberculosis infection is a continuous immune response to *Mycobacterium tuberculosis* antigens, but without evidence of active tuberculosis. Two tests, the tuberculin skin test (TST) and the interferon-gamma release assay (IGRA), are used to screen for latent tuberculosis infection. The IGRA is recommended over the TST for the diagnosis of latent tuberculosis infection in individuals with low-to-intermediate risk of progression to active disease, whereas the IGRA, TST, or dual testing (if the first test is negative) is recommended in patients with a higher risk of developing active tuberculosis (33). The IGRA is frequently favored in developed countries with low disease prevalence because of its more reliable results in patients with previous Bacille Calmette-Guérin vaccination and/or in those receiving corticosteroid treatment.

Before ICIs are initiated, some researchers suggest that an IGRA be performed (31, 34, 35). Varying survival expectancy associated with various types of tumors, differences in the underlying characteristics of patients, risks associated with the cancer itself, and concomitant or previous therapies all undermine the ability to determine the precise risk of developing active tuberculosis associated with ICI therapies (32). A nationwide study in South Korea did not detect increased risk of developing active tuberculosis in patients treated with ICIs compared with the risk in other cancer patients (36). Screening for latent tuberculosis infection is currently not recommended in the general cancer population (22, 23). Therefore, latent tuberculosis infection screening is indicated only in patients with additional risk factors, such as high-risk neoplasms (hematological, head and neck, or lung cancers), other predisposing comorbidities, and/or estimated long survival.

In patients who require anti-TNF-α therapy, the risks and benefits of latent tuberculosis infection screening should be carefully assessed, and different options should be considered. Most guidelines recommend screening because of the significantly increased risk of tuberculosis reactivation in patients receiving these agents for a wide array of inflammatory conditions (30, 37). Screening may also be considered in patients who need high-dose corticosteroids in settings with a high tuberculosis prevalence.

#### Managing latent tuberculosis infection

Treatment for latent tuberculosis infection should be considered in those with a positive test. The potential harms and benefits of treatment for latent tuberculosis infection need to be weighed on an individual basis, accounting for potential pharmacological interactions, the risk of hepatotoxicity, and expected survival. An assessment with an infectious disease specialist and clinical monitoring during treatment are advisable. It is generally accepted that initiation of ICIs should be delayed about 2 weeks after the initiation of antituberculous treatment, in order to improve tolerance and minimize the possibility of immune reconstitution symptoms (32).

#### Diagnosing and treating active tuberculosis in patients receiving ICIs

Active tuberculosis may develop in a patient receiving ICIs. Diagnosis may be complicated by the lack of specificity of signs and/or symptoms, which may mimic oncological disease progression or pseudoprogression, bacterial or fungal infection, or pulmonary irAEs. Therefore, high clinical suspicion is key to an accurate diagnosis. Microbiological confirmation through invasive or non-invasive samples is paramount and necessary in guiding adequate antimycobacterial therapy, which must be weighed according to the characteristics of the patient and potential pharmacological interactions and toxicities. Liver inflammation in the course of treatment must also be carefully assessed, since it may represent toxicity caused by antituberculous therapy or an irAE or be associated with the underlying disease. It is generally supported that ICIs should be withheld during active infection for 2–4 weeks because of the possibility of an exaggerated inflammatory response (32).

### Conclusions

In conclusion, although the use of ICIs has been linked to the development of tuberculosis, the precise risk of this association has not been established. Current evidence does not clearly support routine latent tuberculosis infection screening in these patients. Treatment for latent tuberculosis infection or active tuberculosis should be individually evaluated.

## Immune checkpoint inhibitors in patients with underlying autoimmune diseases

About 3–5% of the world's population has an autoimmune disorder (38–41). Autoimmune and chronic inflammatory diseases have been significantly associated with increased risk of cancer (42). Between 10 and 30% of patients with cancer have one of the more than 80 different autoimmune diseases, either localized in an individual organ or with a systemic presentation. Patients with cancer and autoimmune diseases have shorter survival durations, poorer quality of life, and higher health care costs than do cancer patients without autoimmune diseases (43–45).

Cancer patients with autoimmune diseases have been excluded from most ICI trials because of concerns about increasing their risk of irAEs and/or flares of their concomitant autoimmune disease. The exact pathophysiology of irAEs is not known and may vary across toxicity phenotypes, but it is attributed to the expansive upregulation of immune pathways caused by ICIs, resulting in inflammatory and autoimmune manifestations that can affect almost any system or organ and can be severe. Although numerous reports have been published describing the occurrence of irAEs and flares in patients with autoimmune diseases [“who are treated with ICIs for cancer”?], most are retrospective in nature. To date, no controlled trial data exist regarding the safety and efficacy of ICIs in patients with cancer and autoimmune disease. Here, we review the evidence of relevant observational data to provide a comprehensive summary of the occurrence of irAEs and autoimmune disease flares and of cancer response to ICIs in patients with preexisting autoimmune disease. Data are still needed on the incidence of irAEs in patients with active vs. stable autoimmune disease and on the use of DMARDs (disease-modifying antirheumatic drugs)/steroids at the initiation of ICI therapy and per ICI used (anti-PD1, antiPDL1, anti-CTLA4).

### Occurrence of irAEs and flares in patients with preexisting autoimmune diseases

Previously, a review of 123 patients whose cases were described in 49 publications reported that in 92 (75%) of these cases, there was an exacerbation of autoimmune disease, irAEs, or both with ICI treatment. The large majority of patients in the review had melanoma (46). However, pooled data from 11 case series (47–57) suggested that the number of patients experiencing any type of irAEs (flares or *de novo*) was 55% [95% confidence interval (CI), 44–66%] (58). For flares, the pooled frequency was 29% (95% CI, 11–49%) and for *de novo* irAEs it was 30% (95% CI, 24–35%). When categorized by type of ICI, 37% (95% CI, 25–50%) of the patients who received anti-PD-1/PD-L1 agents had autoimmune flares, compared with 29% (95% CI, 11–49%) who received anti-CTLA4. Flares were more commonly reported in patients with arthritis (rheumatoid, chronic unspecified, or inflammatory) (33%) and psoriasis (20%).

### Risk of irAEs in patients with and without autoimmune disease

Although evidence from a report in 2017 (47) suggested that the risk of developing an irAE over 2 years of follow-up after initiation of ICIs in patients with autoimmune diseases was 1.5 times higher than that in patients without an autoimmune disease (95% CI, 1.1–2.2), another study in 2019 (59) reported no statistically significant increase in the risk of grade 3 or 4 irAEs, suggesting that the increased risk observed may be limited to grade 1 or 2 toxicities. A similar risk of developing any type of irAE was reported for patients with autoimmune diseases when compared with patients without autoimmune disease who had developed an irAE after exposure to ipilimumab (47).

One study compared the flare rates in patients with autoimmune rheumatologic diseases to rates in patients with autoimmune non-rheumatologic diseases. Patients who had a rheumatologic disease were 4.1 times more likely to develop a flare (95% CI, 1.3–13.4) (56). However, for patients with stable autoimmune disease at the start of ICI therapy, the flare rates were lower (18%) than those of patients with uncontrolled disease (50%) (55).

### Cancer response to ICI in patients with cancer and preexisting autoimmune diseases

The presence of preexisting autoimmune disease was not associated with cancer outcomes such as progression-free survival and overall survival in a systematic review of observational studies (47–49, 52–55, 57, 58). The pooled proportion of patients with cancer and autoimmune disease with complete response after treatment with any ICI was 6% (95% CI, 0–18%) (47–58). The pooled proportion of patients with partial response was 25% (95% CI, 15–36%), with stable disease was 21% (95% CI, 10–34%), and with progressive disease was 46% (95% CI, 31–61%) (58).

The pooled frequency of permanent discontinuation of the ICI was 12% (95% CI, 4–24%) and of temporary discontinuation was 9% (95% CI, 2–18%) (58). Pooled mortality was 31% (95% CI, 11–56%), although none of the deaths were related to the autoimmune disease (47, 49, 52, 53, 55, 56, 58). Death rates were lower in patients with autoimmune disease who developed a flare compared with those with no flares (58).

### Recommendations

The European Society of Medical Oncology has proposed a two-step approach for the care of patients with cancer and underlying autoimmune disease who are considering ICIs. The first step consists of a short-term prevention strategy in which non-immunosuppressant agents are discontinued and replaced with a first-line immunosuppressive or more targeted treatments as opposed to systemic immunosuppression. It is preferable that the autoimmune disease be controlled for 2–4 weeks before ICIs are started. For patients with a rapid disease course, immunosuppressants and ICIs can be introduced simultaneously. Once therapy with ICIs has commenced, close monitoring to manage any potential flares is imperative. Finally, the guidelines recommend maintaining immunosuppressants for the duration of ICI therapy to avoid severe flares (60). In addition, the National Comprehensive Cancer Network recommends the involvement of a multidisciplinary team that includes an autoimmune disease specialist in the decision to initiate ICIs and, when possible, the avoidance of combination therapy with PD1/PD-L1 and CTLA-4 agents (61).

### Conclusions

In conclusion, immune checkpoint inhibition in patients with known autoimmune diseases is possible but requires careful monitoring. Several studies across the globe have reported the use of ICIs in patients with cancer and autoimmune disease in whom the rates of flares and *de novo* irAEs were substantial. Partial response is achieved by at least a quarter of patients with advanced-stage cancer, and permanent discontinuation of the ICI is needed in only a few patients with cancer and autoimmune disease. Therefore, the risk-benefit ratios of immunotherapy in patients with preexisting autoimmune diseases need to be carefully discussed with patients.

## Author contributions

JA-C, ML-O, and IR-C contributed equally to the conception and design of the article, divided the sections, were responsible for one of the three sections, revised and interpreted critically the relevant literature for her/his section and drafted it, and finally reviewed the complete final manuscript. All authors contributed to the article and approved the submitted version.

## Funding

ML-O's work is supported by the National Cancer Institute (#CA237619).

## Conflict of interest

ML-O reported receiving grants from the National Cancer Institute and the Rheumatology Research Foundation outside the submitted work. The remaining authors declare that the research was conducted in the absence of any commercial or financial relationships that could be construed as a potential conflict of interest. The reviewer EMC declared a shared affiliation with the author(s) JA-C and IR-C to the handling editor at the time of review.

## Publisher's note

All claims expressed in this article are solely those of the authors and do not necessarily represent those of their affiliated organizations, or those of the publisher, the editors and the reviewers. Any product that may be evaluated in this article, or claim that may be made by its manufacturer, is not guaranteed or endorsed by the publisher.
