# Supplementary material for: Multidisciplinary approach to treatment with immune checkpoint inhibitors in patients with HIV, tuberculosis, or underlying autoimmune diseases
Source: Front Med (Lausanne). 2022 Jul 15;9:875910. doi: 10.3389/fmed.2022.875910 (PMC9334667; doi:10.3389/fmed.2022.875910)
Supplement: Supplementary file 1 [file Table_1.pdf]

Table S1. Summary of the potential complications and recommendations for each risk group

**Risk group**	**Potential challenges**	**Suggestions for management**
HIV	Impairment of viral control	Initiate ART treatment and achieve viral suppression before the initiation of ICI<br>Do not interrupt ART during ICI treatment<br>Follow-up by an infectious disease specialist is mandatory
LTBI	Tuberculosis reactivation/flare	Screening for LTBI not routinely recommended in absence of additional tuberculosis reactivation risk factors (e.g., anti-TNF-α therapy)<br>If LTBI is diagnosed, consider initiating treatment at least 2 weeks before ICI<br>In patients with untreated LTBI, monitor possible active tuberculosis symptoms
Active tuberculosis	Diagnostic delay due to unspecific symptoms<br>Worsening course of infection	Maintain high clinical suspicion and initiate diagnostic workup if indicated<br>Consider temporally interrupting ICI treatment in cases with high symptom burden
Previous autoimmune disease	Possible autoimmune disease flare	Preferably use first-line immunosuppressive agents or targeted treatments<br>Initiate ICI only if autoimmune disease is controlled<br>Close follow-up for potential flare symptoms is needed<br>Consider avoiding potent ICI therapy (e.g., ICI combination regimens)

ART, antiretroviral treatment; HIV, human immunodeficiency virus; ICI, immune checkpoint inhibitor; LTBI, latent tuberculosis infection; TNF-α, tumor necrosis factor α
